# Supplementary material for: Association between oral microbiome and seven types of cancers in East Asian population: a two-sample Mendelian randomization analysis
Source: Front Mol Biosci. 2023 Nov 21;10:1327893. doi: 10.3389/fmolb.2023.1327893 (PMC10702768; doi:10.3389/fmolb.2023.1327893)
Supplement: Supplementary file 1 [file DataSheet1.ZIP › Supplementary Materials/MR plots of saliva/saliva═╝/Lung Cancer/pheno.858_to_lung cancer_leave_one_out.pdf]

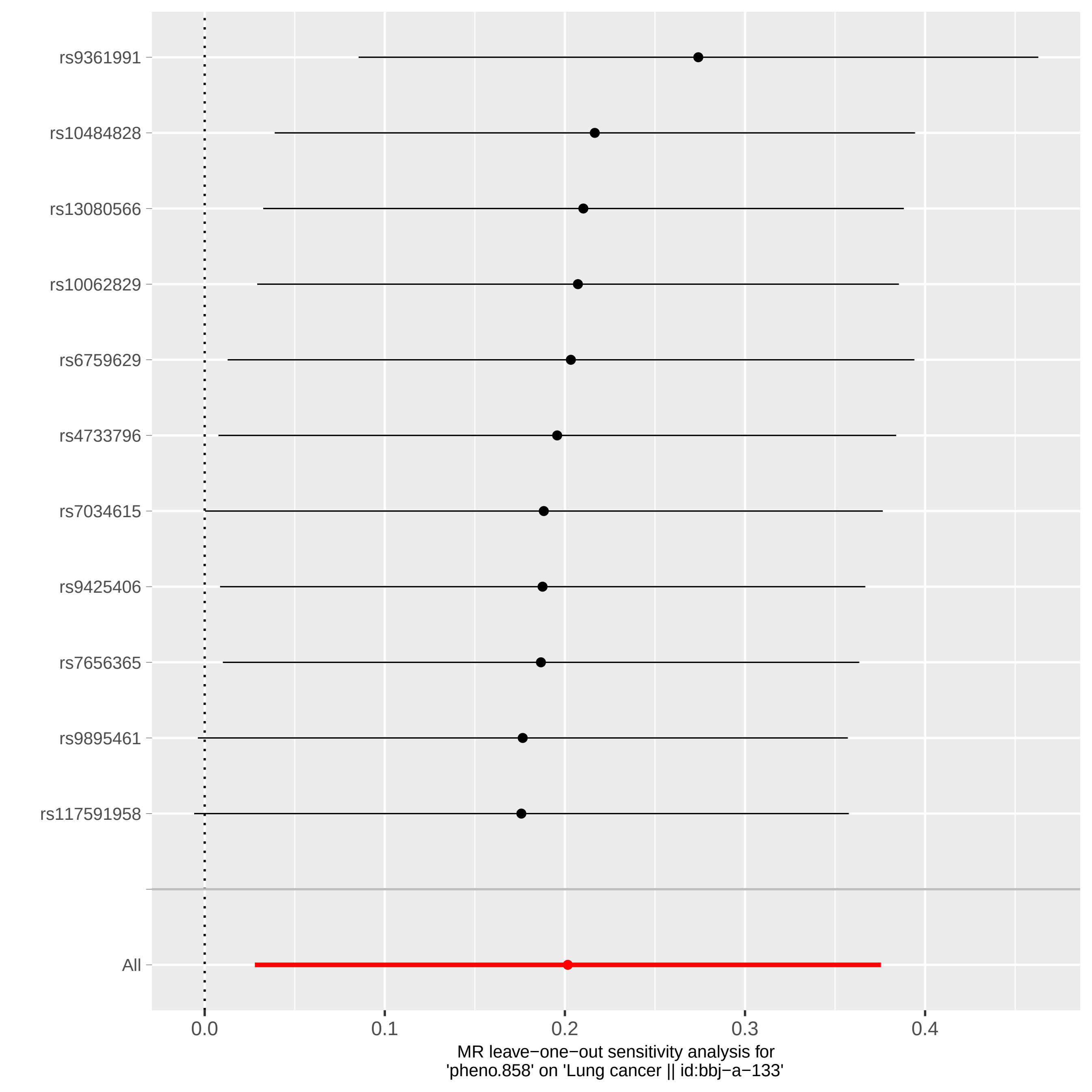
rs9361991
rs10484828
rs13080566
rs10062829
rs6759629
rs4733796
rs7034615
rs9425406
rs7656365
rs9895461
rs117591958
All
0.0
0.1
0.2
0.3
0.4
MR leave−one−out sensitivity analysis for
'pheno.858' on 'Lung cancer || id:bbj−a−133'
